# Overexpression of *LiDXS* and *LiDXR* From Lily (*Lilium* ‘Siberia’) Enhances the Terpenoid Content in Tobacco Flowers

**DOI:** 10.3389/fpls.2018.00909

**Published:** 2018-07-09

**Authors:** Tengxun Zhang, Ming Sun, Yanhong Guo, Xuejun Shi, Yongjuan Yang, Juntong Chen, Tangchun Zheng, Yu Han, Fei Bao, Sagheer Ahmad

**Affiliations:** Beijing Key Laboratory of Ornamental Plants Germplasm Innovation & Molecular Breeding, National Engineering Research Center for Floriculture, Beijing Laboratory of Urban and Rural Ecological Environment, Key Laboratory of Genetics and Breeding in Forest Trees and Ornamental Plants of Ministry of Education, School of Landscape Architecture, Beijing Forestry University, Beijing, China

**Keywords:** *Lilium*, *LiDXS*, *LiDXR*, monoterpenes biosynthesis, floral scent, volatile

## Abstract

*Lilium*, the famous and significant cut flower, emits a variety of volatile organic compounds, which mainly contain monoterpenes, such as myrcene, (E)-β-ocimene, and linalool. To understand the molecular mechanism of monoterpene synthesis in *Lilium*, we cloned two potential genes in the methylerythritol 4-phosphate pathway, namely *LiDXS* and *LiDXR*, from the strong-flavored oriental *Lilium* ‘Siberia’ using a homology-based PCR strategy. The expression levels of *LiDXS* and *LiDXR* were consistent with the emission and accumulation of monoterpenes in different floral organs and during the floral development, indicating that these two genes may play key roles in monoterpene synthesis. Subcellular localization demonstrated that LiDXS and LiDXR are expressed in the chloroplasts. Ectopic expression in transgenic tobacco suggested that the flowers of *LiDXS* and *LiDXR* transgenic lines accumulated substantially more diterpene, sclareol, compared to the plants transformed with empty vector. Surprisingly, increased content of the monoterpene, linalool and sesquiterpene, caryophyllene, were detected in the *LiDXR* transgenic lines, whereas the emission of caryophyllene, increased in one of the *LiDXS* transgenic tobacco lines, indicating that these two genes play significant roles in the synthesis of floral volatiles in the transgenic plants. These results demonstrate that *LiDXR* can contribute to monoterpene biosynthesis in *Lilium* ‘Siberia’; however, the role of *LiDXS* in the biosynthesis of monoterpenes needs further study.

## Introduction

Fragrance is a significant and important character of horticultural plants that provides pleasure and enhances the ornamental value of flowers. Moreover, the aromatic compounds and essential oils present in flowers determine their economic value (Feng et al., 2014). Floral scent is produced by volatile organic compounds (VOCs) with low molecular weight (Pichersky et al., 2006), which are categorized into three major groups, namely terpenoids, phenylpropanoids/benzenoids, and fatty acid derivatives, according to their biosynthetic origin (Muhlemann et al., 2014). Floral VOCs are signals between plants and insects (Piechulla and Pott, 2003; Reinhard et al., 2004; Cseke et al., 2007), and they play vital roles in attracting the pollinators (Pellmyr and Thien, 1986) and in defending against pathogens (Ramak et al., 2014). Among the three major groups of VOCs, benzenoids are used mainly for attracting pollinators, whereas terpenoids and benzenoids protect plants from pathogens (Schiestl, 2010). Terpenoids are the largest class of floral volatiles derived from two common and inter-convertible five-carbon precursors, isopentenyl diphosphate (IPP) and dimethylallyl diphosphate (DMAPP) (McGarvey and Croteau, 1995). Unlike most other organisms, plants use at least two metabolic pathways to synthesize IPP and DMAPP. These include the cytosolic mevalonate (MVA) pathway, which occurs in the cytoplasm and the 2-C-methyl-D-erythritol-4-phosphate (MEP) pathway, which operates in the plastids (Jadaun et al., 2017). It has been shown that volatile monoterpenes and diterpenes, carotenoids, and chlorophylls are all formed by the MEP pathway, whereas the MVA pathway is mainly responsible for the synthesis of volatile sesquiterpenes and triterpenes (Lichtenthaler, 1999; Eisenreich et al., 2004; Chaurasiya et al., 2012; Yadav et al., 2014).

The MEP pathway involves seven enzymatic reactions, starting with the condensation of D-glyceraldehyde-3-phosphate (G3P) and pyruvate, which results in the formation of 1-deoxy-D-xylulose-5-phosphate (DXP) and is catalyzed by DXP synthase (DXS) with the assistance of thiamine pyrophosphate (TPP) and Mg^2+^ (Rodriguez-Concepcion and Boronat, 2002). In the second step, DXP reductoisomerase (DXR) synthesizes the intermediate product, MEP, from DXP in the presence of NADPH and Mn^2+^ or Mg^2+^ (Takahashi et al., 1998). Therefore, the conversion from DXP to MEP is considered the committed step in the formation of IPP, an important intermediate substrate in these two pathways.

Many researchers have proposed that DXS and DXR are the potential control points in the synthesis of isopentenes (Harker and Bramley, 1999; Kuzuyama et al., 2000; Carretero-Paulet et al., 2006). To date, multiple *DXS* and *DXR* genes have been isolated and characterized. The *DXS* genes have been reported frequently not only from the model plant, *Arabidopsis thaliana* (Carretero-Paulet et al., 2006), but also from ornamental plants like *Lavandula latifolia* (Munoz-Bertomeu et al., 2006), *Pelargonium* spp.(Jadaun et al., 2017), *Catharanthus roseus* (Chahed et al., 2000), and *Aquilaria sinensis* (Xu et al., 2014) and from crop plants, such as *Solanum lycopersicum* (Lois et al., 2000), *Zea mays* (Cordoba et al., 2011), *Aconitum balfourii* (Sharma et al., 2016), *Tripterygium wilfordii* (Tong et al., 2015), and *Salvia miltiorrhiza* (Zhou et al., 2016). Because of their relationship in catalyzing a subsequent step in the MEP pathway, the *DXRs* are always illustrated together with the *DXS* genes in *A. thaliana* (Carretero-Paulet et al., 2006), *Dendrobium officinale* (Fan et al., 2016), *Rosa rugosa* (Feng et al., 2014), *L*. *latifolia* (Mendoza-Poudereux et al., 2014), *Osmanthus fragrans* (Zeng et al., 2016), and other plants. However, detailed studies are required for understanding whether the *DXS* and *DXR* genes play key roles in monoterpene biosynthesis of essential oils. In *Ocimum basilicum*, the expression levels of *DXS* and *DXR* genes were positively correlated with the yield of monoterpenes (Xie et al., 2008). In spike lavender, *DXS* was reported to play a crucial role in the biosynthesis of the monoterpene precursor, whereas *DXR* was not found to be the rate-limiting enzyme in this pathway (Mendoza-Poudereux et al., 2014). In the flowers of *R. rugosa*, the *RrDXR* gene might play a key role in the biosynthesis of volatile monoterpenes, but *RrDXS* might not be a key gene (Feng et al., 2014). Surprisingly, the abundance of *DXS* and *DXR* transcripts in *O. fragrans* flowers was inconsistent with the emission of monoterpenes (Zeng et al., 2016). Whether *DXS* and *DXR* genes play important roles in monoterpene synthesis in lily, needs further investigation.

Lily, a popular and commercially important cut flower, emits significant amounts of VOCs, consisting mainly of monoterpenes (Hu et al., 2017; Kong et al., 2017). Although the floral scents of the oriental *Lilium* hybrids are flowery and sweet, it is not liked by people owing to the emission of large amounts of volatile compounds (Johnson et al., 2016). To improve this aspect and to produce new *Lilium* varieties with fragrance emission within the acceptable range, it is important to understand the biosynthesis of monoterpenes in *Lilium* in detail. However, until date the molecular mechanism of monoterpene biosynthesis in lily is poorly understood.

In the present study, we investigated the two potential rate-limiting enzymes, *LiDXS* and *LiDXR*, in the strong-scented oriental lily, *Lilium* ‘Siberia’, to improve our understanding of the process of synthesis volatile compounds in floral organs. Considering the fact that *Lilium* is a model plant used for understanding the molecular pathway related to floral scents (Johnson et al., 2016), our elucidation of the monoterpene metabolic pathway in lily provides the theoretical basis for future improvement of its flowering traits through genetic engineering, and lays a foundation for genetic improvement and molecular breeding for floral traits of ornamental plants.

## Materials and Methods

### Plant Material

*Lilium* ‘Siberia’ was collected from Xiaotangshan, Beijing. Healthy buds of consistent size, prior to the budding stage, were cut and water-cultured in a condition-stable phytotron at 25 ± 1°C under 55% relative humidity and a photoperiod of 16 h light and 8 h dark.

### RNA Extraction and Gene Cloning

The inner petals from at least three flowers in full-bloom on day 2 were collected at 9:00 h and were immediately frozen in liquid nitrogen and stored in a -80°C freezer for RNA extraction. The total RNA was extracted using Trizol reagent (Invitrogen, Carlsbad, CA, United States) according to the manufacturer’s instructions. The total RNA was digested at 37°C with RQ1 RNase-free DNase (Promega, United States) to eliminate the residual genomic DNA. To synthesize the first-strand cDNA, 5 μg total RNA was transcribed using GoScript^TM^ Reverse Transcription System (Promega, United States) as per the prescribed protocol. To obtain an intermediate nucleotide fragment of *LiDXS*, a pair of degenerate primers, DXS-F1 and DXS-R1, was designed by aligning the conserved domains of different DXS protein sequences retrieved from National Center for Biotechnology Information (NCBI) (Accession Nos. BAD43377.1, ACF60511.1, NP_001234672.1, ACT32136.1, and AEZ53173.1). We obtained the first-strand cDNA for 3′- and 5′-rapid amplification of cDNA ends (RACE) as per the procedure described in the user manual of SMARTer^®^ RACE 5′/3′ Kit, and designed the gene-specific primers, 3′ GSP and 5′ GSP for amplifying the 3′- and 5′-ends. The intermediate fragment and the 3′- and 5′-ends were assembled to determine the full-length sequence of *LiDXS*. Subsequently, we designed another pair of primers, DXS-F2 and DXS-R2, to amplify the open reading frame (ORF) sequence.

The ORF of *DXR* was amplified using the primers, DXR-F and DXR-R, designed on the basis of the sequence of this gene from *Lilium longiflorum* (Accession No. KF765491). All the PCR products were sub-cloned into pCloneEZ-*TOPO* vector; the recombinant vector was transformed into *Escherichia coli* DH5α and the positive clones were screened by sequencing. The sequences of the primers used in this study are listed in Supplementary Table 1.

### Bioinformatics Analysis

The ClustalX 2.0 (Larkin et al., 2007) software was used for alignment of the sequences from *L.* ‘Siberia’ and other monocot, eudicot, and protist species. The multiple alignments of sequences were edited using the BioEdit 7.0 (Hall, 1999). MEGA 5.0 was used to construct the phylogenetic tree using the protein sequences from different species based on the neighbor-joining method with 1000 bootstrap replicates. The subcellular localization of the two potential genes was conducted using Plant-mPLoc, ChloroP, and WoLF PSORT online tools.

### Subcellular Localization

The vector construction was done using the In-Fusion^®^ HD Cloning Kit (Clontech, Japan) following the recommended protocol. The ORFs of *LiDXS* and *LiDXR* were amplified using gene-specific primers (Supplementary Table 1) with 15-bp homologous sequences with plant expression vector *pSuper1300::GFP* containing the recognition sites for *Xba*I and *Spe*I. The vector *pSuper1300::GFP* was linearized by digestion with *Xba*I and *Spe*I. The targeted gene fragments and vectors were ligated by the In-fusion enzyme to construct the recombinant plasmids, *pSuper1300::LiDXS::GFP* and *pSuper1300::LiDXR::GFP*, driven by the CaMV-35S promoter. The recombinants were transformed into *Agrobacterium tumefaciens* GV3101 using freeze-thaw method for further study. The final OD_600_ of the *Agrobacterium* cells transformed with the construct re-suspended in 5 mL of infiltration buffer (10 mM MgCl_2_, 10 mM MES-KOH; pH 5.6–5.7) was adjusted to 0.5. The *Agrobacterium* solution containing 1/1000 volume of acetosyringone (150 mM) was incubated without shaking at 28°C for 2 h before injecting the leaves. The underside of the leaves of *Nicotiana benthamiana* was injected using a syringe and the infected leaves were exposed to long daylight for 48–56 h. The labeled pieces of leaves (1 cm in diameter) were placed on the glass slide. The expression of green fluorescent protein (GFP) was observed under the confocal laser scanning microscope, Leica TCS SP8 (Leica, Germany).

### Gene Expression Analysis

To determine the levels of the *LiDXS* and *LiDXR* transcripts during the flowering stages and in different floral tissues, quantitative real-time PCR (qRT-PCR) was performed using the PikoReal real-time PCR system (Thermo Fisher Scientific, China). Each sample was pooled in at least three replicates and the three pools served as the three biological replicates; for each replicate, qRT-PCR was performed thrice. Only the inner petals were collected from 13:00 to 15:00 h at every stage. RNA extraction was performed as described above and the cDNA was synthesized according to the instructions in the manual of PrimeScript^TM^ RT reagent Kit with gDNA Eraser (Perfect Real Time) (TaKaRa, Japan). The PCRs were carried out using cDNA diluted 30-times with the SYBR^®^ Premix Ex Taq^TM^ Mix II (TaKaRa, Japan). The procedure for qRT-PCR was the classical three-step method and the relative transcript levels were computed using the 2^-ΔΔ^*^Ct^* method, as described previously (Zhang et al., 2017). The *Actin* gene (Accession number: AB438963) was used as the internal reference, as described by earlier (Zhang et al., 2017). The sequences of all the gene-specific primers are listed in Supplementary Table 1.

### Transformation and Screening of Tobacco

The *pSuper1300::LiDXR* and *pSuper1300::LiDXS* constructs used for the stable transformation of tobacco were generated as described above except for the linearized plant expression vector, *pSuper1300*, without the GFP label.

They were transformed into wild type tobacco (*Nicotiana tabacum* ‘NC89’) leaf disks through *A. tumefaciens* GV3101-mediated freeze-thaw transformation. For obtaining the transgenic lines, *Agrobacterium*-mediated transformation of tobacco leaf disks was performed as described previously (Burow et al., 1990). The positive transgenic plants were screened on Murashige and Skoog’s (MS) medium supplemented with 50 mg/L hygromycin, and were identified through PCR. The T1 and T2 transgenic seeds were collected. All the T2 seeds (T3) were generally transgenic and could be used for the determining the content of the floral volatiles. All the transgenic plants were cultured under the same conditions with 16 h light at 24°C /8 h night at 18°C.

For analyzing the expression levels of *LiDXS* and *LiDXR* in the transformed tobacco plants, we collected the flowers at 9:00 h on the first day of flowering. For each line, at least three petals from the transgenic tobacco flowers were pooled and real-time PCR was performed following the steps described above using *NtEFα1* as the endogenous control gene. The expression levels of endogenous *NtDXS* and *NtDXR* genes in tobacco were detected as well. The sequences of all the gene-specific primers are listed in Supplementary Table 1.

### Terpenoid Quantification in Tobacco

The floral volatiles in the transgenic tobacco plants were detected mainly by using headspace solid-phase microextraction (HS-SPME). Each sample, collected from three tobacco flowers, was put in the headspace bottle, and the samples from each line were taken in triplicates. The fiber (50/30 μm DVB/CAR/PDMS) was conditioned for 2 h at 250°C in the gas chromatography inlet before the first volatile extraction. The sample was equilibrated for 3 h at 25°C and collection was done at 50°C for 30 min using the extractor inserted into the upper side of the vial. Immediately after extraction, the fibers were desorbed for 5 min at 250°C in the injection port and then gas chromatography-mass spectrometry (GC/MS) analysis was performed. GC/MS was carried out using Shimadzu CGMS-QP2010 (Shimadzu, Kyoto, Japan) equipped with a DB-5MS capillary column (30 m × 0.25 mm × 0.25 μm, Shimadzu). The chromatography program started at 40°C for 2 min, and the temperature was increased to 200°C at a rate of 5°C/min and held at this temperature for 6 min; the inlet temperature was set at 200°C, total flow was 27.5 mL/min, and the split ratio was 20. The carrier gas used was helium. The parameters for mass spectrometer were as follows: the electron potential was set to 1 KV, the mass scan range (m/z) was 30–300 amu, and the ion source temperature was set to 250°C. The volatile compounds in the flowers were identified by comparison with the compounds present in the National Institute of Standards and Technology (NIST) 11 library using GC/MS Postrun Analysis software. The relative content of each compound was calculated based on the peak area normalization method.

### Statistical Analysis

The statistical significance was determined with SPSS 22.0 (SPSS, Inc., Chicago, IL, United States) using one-way ANOVA. The standard deviation was calculated using triplicate samples and significant differences (*p* < 0.05) between the transformed lines and transgenic tobacco transformed with the empty vector were compared. The bar charts representing the data were drawn with Origin Pro 8.0 (Northampton, MA, United States).

## Results

### Characterization of *LiDXS* and *LiDXR*

**FIGURE 1:**
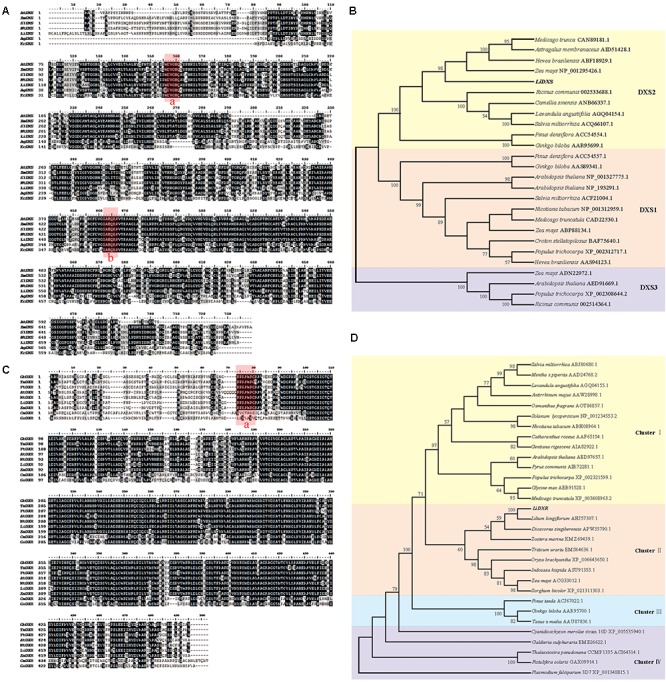
Multiple alignment and phylogenetic analysis of protein sequence of DXS and DXR from different species. **(A)** Alignment of deduced amino acid sequences of DXS proteins performed using Cluster X and DNAMAN 7.0. Species abbreviations in gene names are as follows: At: *Arabidopsis thaliana*, Zm: *Zea mays*, Sl: *Solanum lycopersicum*, Nt: *Nicotiana tabacum*, Ag: *Agrobacterium tumefaciens* (AIC32317.1), Ec: *Escherichia coli* (EGT70614.1). Their accession numbers were in parallel with DXS2 protein in **(B)**. Identical amino acid residues are shaded black, and the similar ones are shaded gray. The highly conserved domains were marked red: box a is WDVGHM motif, box b is IAEQHA motif. **(B,D)** Phylogenetic tree of DXSs and DXRs proteins, respectively, using neighbor-joining method with 1000 bootstrap replicates and showing values more than 50%. Accession numbers of the amino acid sequences accession numbers registered in NCBI were listed after the species names. **(C)** Alignment of amino acid sequences of LiDXR and DXRs proteins from other species. Species abbreviations in gene names are as follows: Gb: *Ginkgo biloba*, Tm, *Taxus* ×*media*, Pt: *Pinus taeda*, At: *Arabidopsis thaliana*, Nt: *Nicotiana tabacum*, Zm: *Zea mays*, Cm: *Cyanidioschyzon merolae* strain 10D, Gs: *Galdieria sulphuraria*. The assession number of these species were the same as that in part D. The P(P/Q)PAWPG(R/T) motif was labeled in box a.

To identify the function of *LiDXS* and *LiDXR* in terpene biosynthesis, we isolated their ORFs using homologous cloning from *L.* ‘Siberia.’ The full-length cDNA sequence of *LiDXS* (Accession No. MF078467) was found to be 2479 bp, containing an ORF of 2199 bp encoding 732 amino acids. The alignment of the protein sequences of DXS from different sources using Clustal X and BioEdit showed that LiDXS was weakly conserved in the N-terminal and contained the typical WDVGHM and IAEQHA domains (**Figure 1A**), which are believed to be closely related to the catalytic function. The phylogenetic analysis was performed based on the selected DXS protein sequences. The results showed that the phylogenetic tree consisted of three clades. In addition, LiDXS belonged to the DXS type-2 (DXS2) clade and had higher homology to DXS2 from *Z. mays* (**Figure 1B**). The ORF length of *LiDXR* (Accession No. MF078468) was 1419 bp, encoding 472 amino acids. The blastp analysis showed that LiDXR contains two putative domains: DXP_reductoisom (78–206 amino acid), which is present on the C-terminus of the pfam02670 domain in bacterial and plant proteins, and DXP_redisom_C (28–472 amino acid). Moreover, the alignment of DXR sequences from eukaryotes and prokaryotes revealed that the DXRs were highly homologous and shared a conserved P(P/Q)PAWPG(R/T) motif (**Figure 1C**), which was not present in the DXR sequences from protists and regarded as a signature for DXR proteins in all plants (Devi et al., 2015). The sequence was not conserved at the N-terminal end (positions 1–54), whereas the extended region (55–77 amino acids) and the C-terminal region were strongly conserved. The phylogenetic tree of DXR sequences revealed that they were organized into four groups: cluster I comprised eudicots, monocots formed cluster II, gymnosperm formed cluster III, and cluster IV comprised protists; the phylogenetic tree revealed the evolutionary relationship among the different species (**Figure 1D**). In lily, the DXR sequence was conserved; for example, the sequence of LiDXR was highly similar to that of DXR from another monocot, *L. longiflorum*.

**FIGURE 2:**
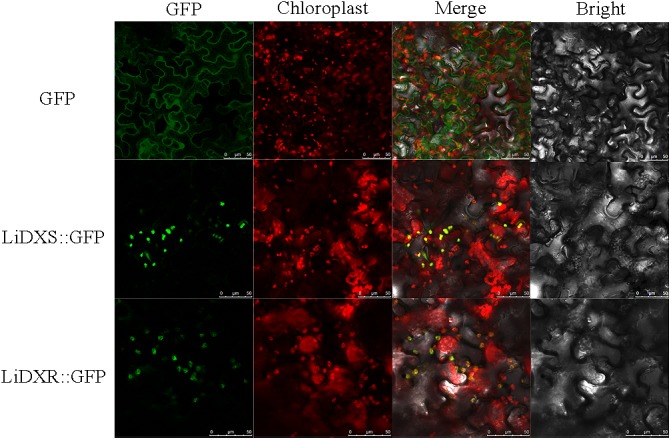
Subcellular localization of LiDXS and LiDXR. The fusion proteins (LiDXS::GFP and LiDXR::GFP) and GFP as a control protein were observed under the confocal laser scanning microscope. The first panel was constructed in the green fluorescence channel. The second panel was the chloroplast autofluorescence channel. The merged images were formed from the first two panels. Bar = 10 μm.

LiDXR and LiDXS were predicted to be localized to the chloroplasts using the online softwares, Plant-mPLoc, WoLF POST, and ChloroP. To determine the intracellular localization and roles of LiDXR and LiDXS, construct vectors and empty vector were transformed into the leaves of *N. benthamiana*. We found that the GFP signal from the empty vector was obtained from the nucleus and cytoplasm, whereas when LiDXS and LiDXR were fused to GFP, the signal was localized in the chloroplasts (**Figure 2**).

### *LiDXS* and *LiDXR* Expression in Floral Organs and Developmental Stages

**FIGURE 3:**
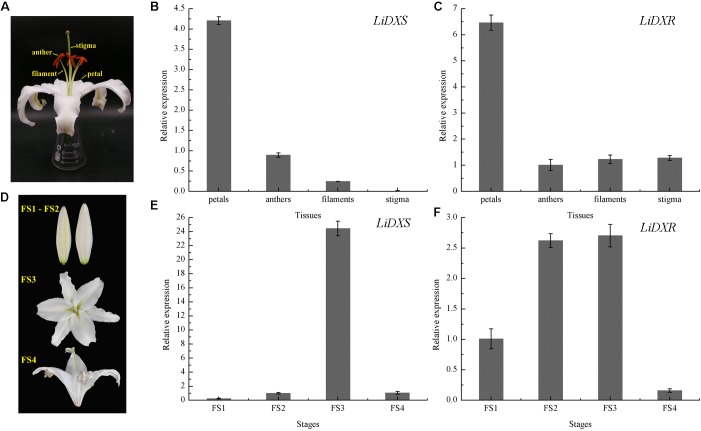
The relative expression levels of *LiDXS* and *LiDXR* genes. **(A)** Petal, filament, anther, and stigma of a flower. **(B,C)** Expression levels of *LiDXS* and *LiDXR* genes in different flower parts at the first full-blooming day, respectively. **(D)** Flower development stages: FS1–FS4. **(E,F)** Expression patterns of *LiDXS* and *LiDXR* genes, respectively, during flower development stages.

To investigate the possible role of *LiDXS* and *LiDXR* in monoterpene production, the expression profiles of these genes was determined in different floral organs and during four flowering stages using qRT-PCR. We gathered the whole flowers on the first day of the full-bloom stage, and divided them into four parts: petals, filaments, anthers, and stigma (**Figure 3A**). The process of flowering was divided into four stages: (FS1) budding stage, (FS2) initial flowering stage, (FS3) full-blooming stage, (FS4) wilting stage (**Figure 3D**). The results indicated that the expression levels of *LiDXS* and *LiDXR* were consistently higher in petals as compared with those in the remaining organs (**Figures 3B,C**). Their expression levels increased from the budding stage to the full-bloom stage and then decreased during the withering stage (**Figures 3E,F**), consistent with the release of floral volatiles in *Lilium* (Hu et al., 2017).

### Overexpression of *LiDXS* and *LiDXR* in Tobacco Affects the Accumulation of Terpenoids

**FIGURE 4:**
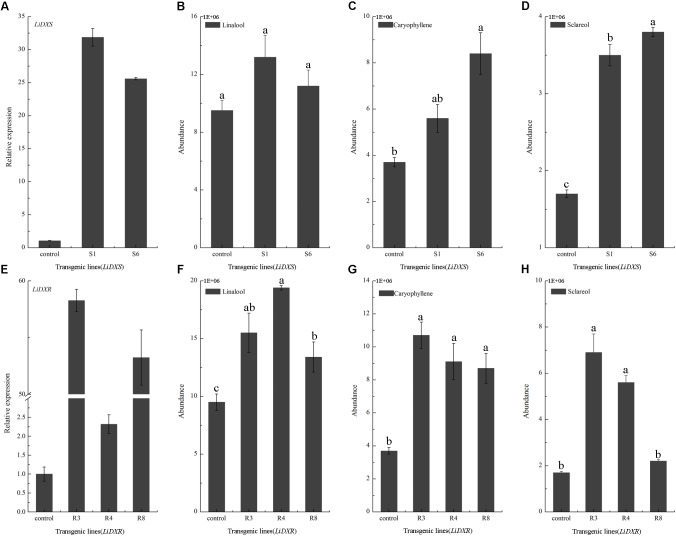
Gene expression levels and volatiles emission amounts in transgenic plants. **(A,E)** The expression levels of *LiDXS* and *LiDXR* genes in different transgenic lines determined by qRT-PCR. *NtEFα1* gene was used as the endogenous control. **(B–D)** Relative emission amounts of linalool, caryophyllene, sclareol in *LiDXS* transgenic lines. **(F**–**H)** Relative emission amounts of linalool, caryophyllene, and sclareol in *LiDXR* transgenic lines. The columns represent average values for each line, and error bars show the standard deviation of three biological replicates. Duncan statistical analysis at the was used for testing significance of the observed differences level of at the *p* < 0.05 level.

**FIGURE 5:**
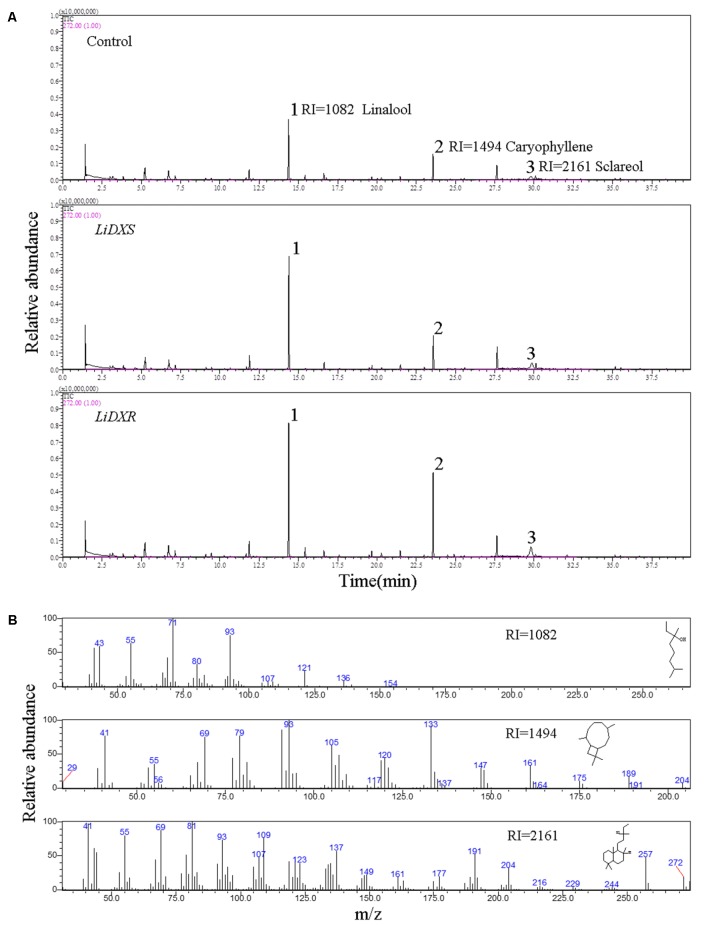
The identification of flower volatiles from transgenic *N. tabacum*. **(A)** GC trace of flower of transgenic *N. tabacum* plants. **(B)** Mass spectra of linalool, caryophyllene, and sclareol. The products were identified by comparison with compounds in the library NIST11.

*LiDXS* and *LiDXR* might be the crucial genes contributing to the biosynthesis of monoterpenes (linalool, (E)-β-ocimene, and myrcene), as revealed by real-time PCR (**Figure 3**). The stably transformed tobacco plants were obtained by infecting leaf disks with *A. tumefaciens* GV3101 carrying *pSuper1300::LiDXR* and *pSuper1300::LiDXS*. At least 30 transgenic tobacco plants (T1) were obtained for each of the target genes. The transformed lines were confirmed by hygromycin resistance and the presence of the target genes in the transgenic plants was determined by PCR. The seeds of T1 plants (T2 seeds) were screened on MS medium containing hygromycin. Two positive transgenic lines harboring *LiDXS* (S1 and S6) and three lines harboring *LiDXR* (R3, R4, and R8) were selected using RT-PCR. These were all provided in Supplementary Figure 1. The floral scent in all these transgenic plants were obviously increased without any changes in the phenotypic characters, such as flower color and shape (Supplementary Figure 2). The transgenic tobacco plants transformed with the empty vector were treated as control. The endogenous and heterogenous expression levels of the transgenes were further confirmed by qRT-PCR. The results showed that the ecpotic expression levels of both the genes were significantly increased in the transgenic lines compared to their levels in the control plants (**Figures 4A,E**). Meanwhile, the endogenous *NtDXS* and *NtDXR* genes in tobacco were expressed stably (Supplementary Figure 3). The floral volatiles in all the lines were examined by HS-SPME-GC-MS. There were no differences in the contents of the volatile compounds between the control and transgenic lines; however, terpenoids were enhanced differently in the transgenic lines (**Figure 5**). In the S1 and S6 lines, the amounts of linalool (a monoterpene) were enhanced slightly but no obvious changes were noticed compared to the level in the control (**Figure 4B**). The content of caryophyllene (a sesquiterpene) was strongly increased in S6, whereas no significant difference was detected in S1 (**Figure 4C**). The content of sclareol (a diterpenoid) was sharply increased in the S1 and S6 lines, and there were significant differences in the content compared to control (**Figure 4D**). The overexpression of *LiDXR* resulted in apparent changes in the contents of different compounds in the transgenic lines. The content of linalool was significantly increased in R3, R4, and R8 lines by 1.6–2.1 times compared to the content in the control plants (**Figure 4F**), and obvious changes in the content of caryophyllene were observed (**Figure 4G**). The content of sclareol was also significantly enhanced in the R3 and R4 lines, whereas no change was detected in the R8 line (**Figure 4H**).

## Discussion

To elucidate the functions of LiDXS and LiDXR, we analyzed these sequences at first. The blastp analysis showed that LiDXS belonged to highly conserved transketolase-like enzyme family, in which the C-terminal domain serves as a regulatory binding site. Both, LiDXS and transketolase, contain histidine residues, which are thought to be involved in proton transport during the reaction (Lois et al., 1998). In addition, they contain a typical conserved motif for the binding of TPP, which acts as a cofactor (Jadaun et al., 2017). The phylogenetic tree of LiDXS revealed that these genes evolved during the evolution of the most advanced angiosperms. The distance of PiDXS from *Pinus densiflora* and GbDXS from *Ginkgo biloba* were the closest; both these plants are gymnosperms. LaDXS, from *Lavandula angustifolia*, clustered together with SaDXS from *S. miltiorrhiza*, both of which belong to Labiatae. LiDXS was next to ZmDXS from *Z. mays*, a monocot. The DXS is reported to be usually encoded by a small family of genes (Walter et al., 2002; Phillips et al., 2008; Cordoba et al., 2009, 2011) and there are many DXS homologs in plants, such as *Z*. *mays* (Cordoba et al., 2011), *Hedychium coronarium* (Yue et al., 2015), and *S. lycopersicum* (Zhou et al., 2016). The three clades of the gene family are differently expressed during plant development and in specific organs (Cordoba et al., 2011), and play different roles in plant development. The first group of *DXS* genes mainly functions as a housekeeping gene (Kim et al., 2005); the second clade is mainly involved in the secondary metabolism of plants (Walter et al., 2002). The third type synthesizes some substances derived from the isoprene pathway, which are necessary but have lesser demand in the plants, such as gibberellins and abscisic acid (Rohmer et al., 1996; Lichtenthaler, 1999). Moreover, in this phylogenetic tree, the proteins are organized into different clades according to their functions, among which the DXS2 clade is involved in the isoprene synthesis pathway; for example, CrDXS participates in the biosynthesis of the monoterpene indole alkaloids in *C*. *roseus* (Burlat et al., 2004). However, the phylogenetic analysis of LiDXR indicates the evolutionary relationship among the DXRs and their diversification, and suggests that the DXRs might have evolved from an ancestral gene (Tong et al., 2015) by parallel duplication (Devi et al., 2015). In lily, LiDXS belonged to the DXS2 clade, suggesting that it might function in the synthesis of monoterpenes.

The enzymes of the MEP pathway were demonstrated to be localized in chloroplasts in soybean, as well as in maize (Cordoba et al., 2011) and *Arabidopsis* (Hsieh et al., 2008), using GFP labeling (Wright et al., 2014). In plant cells, the movement of genes from the photosynthetic endo-symbiont (plastid) to the nucleus mandates that the encoded plastidal proteins must have a transit peptide that can translocate them to their site of function. The plastid localization signals in the DXS and DXR proteins were present on the N-terminus and were not found to be conserved in different species (**Figures 1A,C**). Moreover, the signal peptide was found to be absent in the bacterial DXS and DXR (Carretero-Paulet et al., 2002; Rodriguez-Concepcion and Boronat, 2002). Both LiDXS and LiDXR were predicted to contain a chloroplast transit peptide, respectively, 53 and 48 amino acids in length. The plant DXS proteins contain a typical plastid transit peptide sequence, which determines the plastidal localization of these proteins (Bouvier et al., 1998; Lois et al., 2000). All the enzymatic reactions of the MEP pathway are carried out in the chloroplast and control the metabolic flux in the cells (Hsieh et al., 2008). In the plastid-localized MEP pathway, the DXS enzyme has been demonstrated to be the rate-limiting step; investigations on *Arabidopsis CLA1* mutant, showed that dysfunction of *cla1* (DXS-like gene) results in the lack of isoprenoids leading to albinism (Mandel et al., 1996). The DXS proteins in maize, concluding dxs1, dxs2, and dxs3, were only observed in the chloroplasts (Cordoba et al., 2011). And SmDXS1 and SmDXS2 in *S. miltiorrhiza* were located in the chloroplasts, in agreement with the plastidal operation of the MEP pathway (Zhou et al., 2016). Similar results about DXR genes were found in some plants, such as *Arabidopsis* (Carretero-Paulet et al., 2002) and *Glycine max* (Zhang et al., 2012), providing evidence of plastid localization of plant DXRs. In this study, LiDXS and LiDXR were localized to the chloroplasts providing credence to the proposed roles of LiDXS and LiDXR in the MEP pathway in lily.

Previous studies have shown that the expression of genes for the rate-limiting enzymes controlling the MEP pathway also has features that correspond to the spatial and temporal rhythm of fragrance. For instance, the expression profile of *VvDXS* positively correlates with the accumulation of monoterpenes in *Moscato Bianco* berries (Battilana et al., 2011). Petals are the main organs that release distinct aroma compounds in many plants (Feng et al., 2014). As reported by Feng, the main aromatic components of *R. rugosa* are emitted from petals and stamens. The expression of *RrDXR* in petals was found to be the highest compared to its expression in other organs, such as stamens, pistils, calyxes, and receptacles, whereas the expression of *RrDXS* was lower in petals than in receptacles. This suggests that the isolated *RrDXS* belonged to the DXS1 clade and might not play the main function in the biosynthesis of monoterpenes, whereas *RrDXR* might be playing a key role in the MEP pathway. Here, the expression of *LiDXS* and *LiDXR* were significantly higher in the petals than in the other tissues; this result was consistent with the report that the amounts of volatile compounds from petals were similar to that in the whole detached flower of *L.* ‘Siberia’ (Hu et al., 2017).

The amounts of the emitted floral volatiles exhibited rhythmic patterns during the flowering stages. The analysis of the floral scents from *H. coronarium* indicated that the emission of monoterpenes was gradually enhanced from D1–D3 (budding stage) to D4 (full flowering stage) stage, in which the expression was highest. In addition, transcriptome analysis of samples from different stages, revealed that the expression levels of genes responsible for key enzymes (including *HcDXS2A*, *HcGPPS*, *HcTPS7*, and *HcTPS8*) were the highest at the full flowering stage, indicating that these genes might be involved in the synthesis of floral metabolites (Yue et al., 2015). In our study, the expression levels of *LiDXS* and *LiDXR* were initially lower at the budding stage and were apparently increased at the full flowering stage; subsequently, they were decreased at the wilting stage, as observed in a recent report, in which the terpenoid components peaked at the flowering stage (Hu et al., 2017). The expression levels of *LiDXS* and *LiDXR* were positively correlated with the emission of volatile compounds in *L.* ‘Siberia’, suggesting these two genes might play significant roles in the biosynthesis of monoterpenes.

The DXS and DXR proteins have been proposed to be the key and rate-limiting enzymes of terpenoid biosynthesis in many plants, such as *A. thaliana* (Carretero-Paulet et al., 2006) and *T. wilfordii* (Tong et al., 2015). Increased expression of *DXS* and *DXR* could alter the accumulation of MEP-derived isoprenoids (e.g., chlorophylls and carotenoids) in *A. thaliana* (Carretero-Paulet et al., 2006). *TwDXS1*, *TwDXS2*, and *TwDXR* from *T. wilfordii* promoted the accumulation lycopene in an *E. coli* strain as determined through color complementation assays, indicating that these genes could function in controlling the terpenoid biosynthetic flux (Tong et al., 2015). The overexpression and down-regulation of *SmDXS2* resulted in obvious increase and decrease in the content of tanshinone in transgenic roots of *S*. *miltiorrhiza* (Zhou et al., 2016). Several studies supported the view that DXS and DXR are crucial enzymes in regulating the metabolic flux through the MEP-pathway. In the present study, the high levels of ectopic expression of *LiDXS* and *LiDXR* was responsible for the induction of sclareol biosynthesis. These findings were consistent with a previous study (Morrone et al., 2010), which reported that the overexpression of three genes (*idi*, *dxs*, and *dxr*) led to the greatest increase in the yield of this diterpene. The ectopic expression *AtDXS* gene from *A. thaliana* in *L*. *latifolia* significantly altered the content of monoterpenes in transgenic lavender, without causing changes in growth characteristics and ecological habits (Munoz-Bertomeu et al., 2006). The analysis of the metabolites showed that the content of essential oil (geraniol, linalool, and citronellol) increased in the leaves of rose-scented geranium transiently expressing *LiDXS*. Interestingly, the amount of sesquiterpenes content increased as compared with that in the control (Jadaun et al., 2017), which consistent with the findings in our transgenic lines. The content of caryophyllene was enhanced in the R3, R4, R8, and S6 lines. The tobacco plants transformed with *LiDXR* also accumulated substantial amount of linalool without any changes in the composition of plant volatiles with respect to the plants with empty vector. Similar results have been reported in transgenic peppermint (*Mentha* ×*piperita*) (Mahmoud and Croteau, 2001). Because floral volatile compounds in lily mainly comprise of monoterpenes, such as linalool, (E)-β-ocimene and myrcene (Hu et al., 2017), *LiDXR* is a crucial gene associated with the synthesis of monoterpenes, even more terpenoids, and the function of *LiDXS* in this process needs to be verified.

## Conclusion

This is the first report on the cloning of *LiDXS* and *LiDXR* from oriental *Lilium* ‘Siberia’ based on an approach using homologous cloning and RACE. The expression patterns of *LiDXS* and *LiDXR* were consistent with the emission amounts of volatile compounds, indicating the potential roles of these genes in the biosynthesis of monoterpenes. The subcellular localization revealed that these two crucial enzymes are located in the plastids. Moreover, *LiDXR* was identified to play effective roles in the regulation of flower scents. However, we did not produce sufficient evidence regarding the regulatory role of *LiDXS* in monoterpene biosynthesis in the present study. Therefore, more research is needed to verify the function of *LiDXS*. Our results provide important background information on monoterpene biosynthesis in *Lilium*.

## Author Contributions

TxZ and MS conceived and designed the experiments. YG, YY, and JC prepared the plant materials. TxZ, YG, and XS performed the experiments. TxZ analyzed the data and wrote the paper. YH and TcZ played important roles in interpreting the results. FB provided the vector *pSuper 1300* plasmid and revised the manuscript. SA and TxZ revised the manuscript. MS read and approved the final manuscript.

## Conflict of Interest Statement

The authors declare that the research was conducted in the absence of any commercial or financial relationships that could be construed as a potential conflict of interest.

## References

[B1] Battilana J. Emanuelli F. Gambino G. Gribaudo I. Gasperi F. Boss P. K. (2011). Functional effect of grapevine 1-deoxy-D-xylulose 5-phosphate synthase substitution K284N on Muscat flavour formation. *J. Exp. Bot.* 62 5497–5508. 10.1093/jxb/err231 21868399 PMC3223048

[B2] Bouvier F. Harlingue A. Suire C. Backhaus R. A. Camara B. (1998). Dedicated roles of plastid transketolases during the early onset of isoprenoid biogenesis in pepper fruits. *Plant Physiol.* 117 1423–1431. 10.1104/pp.117.4.1423 9701598 PMC34906

[B3] Burlat V. Oudin A. Courtois M. Rideau M. St-Pierre B. (2004). Co-expression of three MEP pathway genes and geraniol 10-hydroxylase in internal phloem parenchyma of *Catharanthus roseus* implicates multicellular translocation of intermediates during the biosynthesis of monoterpene indole alkaloids and isoprenoid-derived primary metabolites. *Plant J.* 38 131–141. 10.1111/j.1365-313X.2004.02030.x 15053766

[B4] Burow M. Chlan C. Sen P. Lisca A. Murai N. (1990). High-frequency generation of transgenic tobacco plants after modified leaf disk cocultivation with *Agrobacterium tumefaciens*. *Plant Mol. Biol. Rep.* 8 124–139. 10.1007/BF02669766

[B5] Carretero-Paulet L. Ahumada I. Cunillera N. O. Rodríguez-Concepción M. Ferrer A. Boronat A. (2002). Expression and molecular analysis of the Arabidopsis DXR gene encoding 1-deoxy-D-xylulose 5-phosphate reductoisomerase, the first committed enzyme of the 2-*C*-methyl-D-erythritol 4-phosphate pathway. *Plant Physiol.* 129 1581–1591. 10.1104/pp.003798 12177470 PMC166745

[B6] Carretero-Paulet L. Cairo A. Botella-Pavia P. Besumbes O. Campos N. Boronat A. (2006). Enhanced flux through the methylerythritol 4-phosphate pathway in Arabidopsis plants overexpressing deoxyxylulose 5-phosphate reductoisomerase. *Plant Mol. Biol.* 62 683–695. 10.1007/s11103-006-9051-9 16941216

[B7] Chahed K. Clastre M. Oudin A. Guivarch N. Hamdi S. Chénieux J. C. (2000). 1-Deoxy-D-xylulose 5-phosphate synthase from periwinkle: cDNA identification and induced gene expression in terpenoid indole alkaloid-producing cells. *Plant Physiol.* 38 559–566. 10.1016/S0981-9428(00)00781-6

[B8] Chaurasiya N. D. Sangwan N. S. Sabir F. Misra L. Sangwan R. S. (2012). Withanolide biosynthesis recruits both mevalonate and DOXP pathways of isoprenogenesis in Ashwagandha *Withania somnifera* L. (Dunal). *Plant Cell Rep.* 31 1889–1897. 10.1007/s00299-012-1302-4 22733207

[B9] Cordoba E. Porta H. Arroyo A. Roman C. S. Medina L. Rodriguez-Concepcion M. (2011). Functional characterization of the three genes encoding 1-deoxy-D-xylulose 5-phosphate synthase in maize. *J. Exp. Bot.* 62 2023–2038. 10.1093/jxb/erq393 21199890

[B10] Cordoba E. Salmi M. Leon P. (2009). Unravelling the regulatory mechanisms that modulate the MEP pathway in higher plants. *J. Exp. Bot.* 60 2933–2943. 10.1093/jxb/erp190 19584121

[B11] Cseke L. J. Kaufman P. B. Kirakosyan A. (2007). The biology of essential oils in the pollination of flowers. *Nat. Prod. Commun.* 2 1317–1336.

[B12] Devi K. Dehury B. Phukon M. Modi M. K. Sen P. (2015). Novel insights into structure–function mechanism and tissue-specific expression profiling of full-length dxr gene from *Cymbopogon winterianus*. *FEBS Open Bio* 5 325–334. 10.1016/j.fob.2015.04.005 25941629 PMC4412881

[B13] Eisenreich W. Bacher A. Arigoni D. Rohdich F. (2004). Biosynthesis of isoprenoids via the non-mevalonate pathway. *Cell. Mol. Life Sci.* 61 1401–1426. 10.1007/s00018-004-3381-z 15197467 PMC11138651

[B14] Fan H. H. Wu Q. J. Wang X. Wu L. S. Cai Y. P. Lin Y. (2016). Molecular cloning and expression of 1-deoxy-D-xylulose-5-phosphate synthase and 1-deoxy-D-xylulose-5-phosphate reductoisomerase in *Dendrobium officinale*. *Plant Cell Tissue Org.* 125 381–385. 10.1007/s11240-016-0945-1

[B15] Feng L. G. Chen C. Li T. L. Wang M. Tao J. Zhao D. Q. (2014). Flowery odor formation revealed by differential expression of monoterpene biosynthetic genes and monoterpene accumulation in rose (*Rosa rugosa* Thunb.). *Plant Physiol. Biochem.* 75 80–88. 10.1016/j.plaphy.2013.12.006 24384414

[B16] Hall T. A. (1999). BioEdit: a user-friendly biological sequence alignment editor and analysis program for Windows 95/98/NT. *Nucleic Acids Symp. Ser.* 41 95–98.

[B17] Harker M. Bramley P. M. (1999). Expression of prokaryotic 1-deoxy-D-xylulose-5-phosphatases in *Escherichia coli* increases carotenoid and ubiquinone biosynthesis. *FEBS Lett.* 448 115–119. 10.1016/S0014-5793(99)00360-9 10217421

[B18] Hsieh M. H. Chang C. Y. Hsu S. J. Chen J. J. (2008). Chloroplast localization of methylerythritol 4-phosphate pathway enzymes and regulation of mitochondrial genes in *ispD* and *ispE* albino mutants in Arabidopsis. *Plant Mol. Biol.* 66 663–673. 10.1007/s11103-008-9297-5 18236010

[B19] Hu Z. H. Tang B. Wu Q. Zheng J. Leng P. S. Zhang K. Z. (2017). Transcriptome sequencing analysis reveals a difference in monoterpene biosynthesis between scented *Lilium* ‘Siberia’ and Unscented *Lilium* ‘Novano’. *Front. Plant. Sci.* 8:1351. 10.3389/fpls.2017.01351 28824685 PMC5543080

[B20] Jadaun J. S. Sangwan N. S. Narnoliya L. K. Singh N. Bansal S. Mishra B. (2017). Over-expression of *DXS* gene enhances terpenoidal secondary metabolite accumulation in rose-scented geranium and *Withania somnifera*: active involvement of plastid isoprenogenic pathway in their biosynthesis. *Physiol. Plant.* 159 381–400. 10.1111/ppl.12507 27580641

[B21] Johnson T. S. Schwieterman M. L. Kim J. Y. Cho K. H. Clark D. G. Colquhoun T. A. (2016). Lilium floral fragrance: a biochemical and genetic resource for aroma and flavor. *Phytochemistry* 122 103–112. 10.1016/j.phytochem.2015.11.010 26654856

[B22] Kim B. R. Kim S. U. Chang Y. J. (2005). Differential expression of three 1-deoxy-D-xylulose-5-phosphate synthase genes in rice. *Biotechnol. Lett.* 27 997–1001. 10.1007/s10529-005-7849-1 16132843

[B23] Kong Y. Bai J. R. Lang L. X. Bao F. Dou X. Y. Wang H. A. (2017). Variation in floral scent compositions of different lily hybrid groups. *J. Am. Soc. Hort. Sci.* 142 175–183. 10.21273/Jashs03934-16

[B24] Kuzuyama T. Takagi M. Takahashi S. Seto H. (2000). Cloning and characterization of 1-deoxy-D-xylulose 5-phosphate synthase from *Streptomyces* sp. strain CL190, which uses both the mevalonate and nonmevalonate pathways for isopentenyl diphosphate biosynthesis. *J. Bacteriol.* 182 891–897. 10.1128/JB.182.4.891-897.2000 10648511 PMC94361

[B25] Larkin M. A. Blackshields G. Brown N. P. Chenna R. McGettigan P. A. McWilliam H. (2007). Clustal W and Clustal X version 2.0. *Bioinformatics* 23 2947–2948. 10.1093/bioinformatics/btm404 17846036

[B26] Lichtenthaler H. K. (1999). The 1-deoxy-D-xylulose-5-phosphate pathway of isoprenoid biosynthesis in plants. *Annu. Rev. Plant Physiol. Plant Mol. Biol.* 50 47–65. 10.1146/annurev.arplant.50.1.47 15012203

[B27] Lois L. M. Campos N. Rosa-Putra S. Danielsen K. Rohmer M. Boronat A. (1998). Cloning and characterization of a gene from *Escherichia coli* encoding a transketolase-like enzyme that catalyzes the synthesis of D-1-deoxyxylulose 5-phosphate, a common precursor for isoprenoid, thiamin, and pyridoxol biosynthesis. *Proc. Natl. Acad. Sci. U.S.A.* 95 2105–2110. 10.1073/pnas.95.5.2105 9482846 PMC19265

[B28] Lois L. M. Rodríguez-Concepción M. Gallego F. Campos N. Boronat A. (2000). Carotenoid biosynthesis during tomato fruit development, regulatory role of 1-deoxy-D-xylulose-5-phosphate synthase. *Plant J.* 22 503–513. 10.1046/j.1365-313x.2000.00764.x 10886770

[B29] Mahmoud S. S. Croteau R. B. (2001). Metabolic engineering of essential oil yield and composition in mint by altering expression of deoxyxylulose phosphate reductoisomerase and menthofuran synthase. *Proc. Natl. Acad. Sci. U.S.A.* 98 8915–8920. 10.1073/pnas.141237298 11427737 PMC37535

[B30] Mandel M. A. Feldmann K. A. Herrera-Estrella L. Rocha-Sosa M. León P. (1996). CLA1, a novel gene required for chloroplast development, is highly conserved in evolution. *Plant J.* 9 649–658. 10.1046/j.1365-313X.1996.9050649.x 8653115

[B31] McGarvey D. J. Croteau R. (1995). Terpenoid metabolism. *Plant Cell* 7 1015–1026. 10.1105/tpc.7.7.1015 7640522 PMC160903

[B32] Mendoza-Poudereux I. Muñoz-Bertomeu J. Arrillaga I. Segura J. (2014). Deoxyxylulose 5-phosphate reductoisomerase is not a rate-determining enzyme for essential oil production in spike lavender. *J. Plant Physiol.* 171 1564–1570. 10.1016/j.jplph.2014.07.012 25151124

[B33] Morrone D. Lowry L. Determan M. K. Hershey D. M. Xu M. Peters R. J. (2010). Increasing diterpene yield with a modular metabolic engineering system in *E. coli*: comparison of MEV and MEP isoprenoid precursor pathway engineering. *Appl. Microbiol. Biotechnol.* 85 1893–1906. 10.1007/s00253-009-2219-x 19777230 PMC2811251

[B34] Muhlemann J. K. Klempien A. Dudareva N. (2014). Floral volatiles: from biosynthesis to function. *Plant Cell Environ.* 37 1936–1949. 10.1111/pce.12314 24588567

[B35] Munoz-Bertomeu J. Arrillaga I. Ros R. Segura J. (2006). Up-regulation of 1-deoxy-D-xylulose-5-phosphate synthase enhances production of essential oils in transgenic spike lavender. *Plant Physiol.* 142 890–900. 10.1104/pp.106.086355 16980564 PMC1630752

[B36] Pellmyr O. Thien L. B. (1986). Insect reproduction and floral fragrances: keys to the evolution of the angiosperms. *Taxon* 35 76–85. 10.2307/1221036

[B37] Phillips M. A. Leon P. Boronat A. Rodriguez-Concepcion M. (2008). The plastidial MEP pathway: unified nomenclature and resources. *Trends Plant Sci.* 13 619–623. 10.1016/j.tplants.2008.09.003 18948055

[B38] Pichersky E. Noel J. P. Dudareva N. (2006). Biosynthesis of plant volatiles: nature’s diversity and ingenuity. *Science* 311 808–811. 10.1126/science.1118510 16469917 PMC2861909

[B39] Piechulla B. Pott M. B. (2003). Plant scents - mediators of inter- and intraorganismic communication. *Planta* 217 687–689. 10.1007/s00425-003-1047-y 14558525

[B40] Ramak P. Osaloo S. K. Sharifi M. Ebrahimzadeh H. Behmanesh M. (2014). Biosynthesis, regulation and properties of plant monoterpenoids. *J. Med. Plant Res.* 8 983–991. 10.5897/JMPR2012.387

[B41] Reinhard J. Srinivasan M. V. Zhang S. W. (2004). Olfaction: scent-triggered navigation in honeybees. *Nature* 427 411–411. 10.1038/427411a 14749818

[B42] Rodriguez-Concepcion M. Boronat A. (2002). Elucidation of the methylerythritol phosphate pathway for isoprenoid biosynthesis in bacteria and plastids. A metabolic milestone achieved through genomics. *Plant Physiol.* 130 1079–1089. 10.1104/pp.007138 12427975 PMC1540259

[B43] Rohmer M. Seemann M. Horbach S. BringerMeyer S. Sahm H. (1996). Glyceraldehyde 3-phosphate and pyruvate as precursors of isoprenic units in an alternative non-mevalonate pathway for terpenoid biosynthesis. *J. Am. Chem. Soc.* 118 2564–2566. 10.1021/ja9538344

[B44] Schiestl F. P. (2010). The evolution of floral scent and insect chemical communication. *Ecol. Lett.* 13 643–656. 10.1111/j.1461-0248.2010.01451.x 20337694

[B45] Sharma E. Pandey S. Gaur A. K. (2016). Identification and expression analysis of DXS1 gene isolated from *Aconitum balfourii* Stapf. *Acta Physiol. Plant* 38:233 10.1007/s11738-016-2239-y

[B46] Takahashi S. Kuzuyama T. Watanabe H. Seto H. (1998). A 1-deoxy-D-xylulose 5-phosphate reductoisomerase catalyzing the formation of 2-*C*-methyl-D-erythritol 4-phosphate in an alternative nonmevalonate pathway for terpenoid biosynthesis. *Proc. Natl. Acad. Sci. U.S.A.* 95 9879–9884. 10.1073/pnas.95.17.9879 9707569 PMC21430

[B47] Tong Y. R. Su P. Zhao Y. J. Zhang M. Wang X. J. Liu Y. J. (2015). Molecular cloning and characterization of *DXS* and *DXR* genes in the terpenoid biosynthetic pathway of *Tripterygium wilfordii*. *Int. J. Mol. Sci.* 16 25516–25535. 10.3390/ijms161025516 26512659 PMC4632813

[B48] Walter M. H. Hans J. Strack D. (2002). Two distantly-related genes encoding 1-deoxy-D-xylulose 5-phosphate synthases: differential regulation in shoots and apocarotenoid-accumulating mycorrhizal roots. *Plant J.* 31 243–254. 10.1046/j.1365-313X.2002.01352.x 12164805

[B49] Wright L. P. Rohwer J. M. Ghirardo A. Hammerbacher A. Ortiz-Alcaide M. Raguschke B. (2014). Deoxyxylulose 5-phosphate synthase controls flux through the methylerythritol 4-phosphate pathway in Arabidopsis. *Plant Physiol.* 165 1488–1504. 10.1104/pp.114.245191 24987018 PMC4119033

[B50] Xie Z. Kapteyn J. Gang D. R. (2008). Asystems biology investigation of the MEP/terpenoid and shikimate/phenylpropanoid pathways points to multiple levels of metabolic control in sweet basil glandular trichomes. *Plant J.* 54 349–361. 10.1111/j.1365-313X.2008.03429.x 18248593

[B51] Xu Y. H. Liu J. Liang L. Yang X. Zhang Z. Gao Z. H. (2014). Molecular cloning and characterization of three cDNAs encoding 1-deoxy-D-xylulose-5-phosphate synthase in *Aquilaria sinensis* (Lour.) Gilg. *Plant Physiol. Biochem.* 82 133–141. 10.1016/j.plaphy.2014.05.013 24950429

[B52] Yadav R. K. Sangwan R. S. Sabir F. Srivastava A. K. Sangwan N. S. (2014). Effect of prolonged water stress on specialized secondary metabolites, peltate glandular trichomes, and pathway gene expression in *Artemisia annua* L. *Plant Physiol. Biochem.* 74 70–83. 10.1016/j.plaphy.2013.10.023 24269871

[B53] Yue Y. C. Yu R. C. Fan Y. P. (2015). Transcriptome profiling provides new insights into the formation of floral scent in *Hedychium coronarium*. *BMC Genomics* 16:470. 10.1186/s12864-015-1653-7 26084652 PMC4472261

[B54] Zeng X. L. Liu C. Zheng R. R. Cai X. Luo J. Zou J. J. (2016). Emission and accumulation of monoterpene and the key terpene synthase (TPS) associated with monoterpene biosynthesis in *Osmanthus fragrans* Lour. *Front. Plant Sci.* 6:1232. 10.3389/fpls.2015.01232 26793212 PMC4709469

[B55] Zhang M. Li K. Liu J. Y. Yu D. Y. (2012). Identification and differential expression of two isogenes encoding 1-deoxy-D-xylulose 5-phosphate reductoisomerase in *Glycine max*. *Plant Biotechnol. Rep.* 6 363–371. 10.1007/s11816-012-0233-4

[B56] Zhang T. X. Sun M. Li L. L. Guo Y. H. Xie X. H. Hu B. W. (2017). Molecular cloning and expression analysis of a monoterpene synthase gene involved in floral scent production in lily (*Lilium* ‘Siberia’). *Russ. J. Plant Physiol.* 64 600–607. 10.1134/S1021443717040203 29166855

[B57] Zhou W. Huang F. F. Li S. Wang Y. Zhou C. C. Shi M. (2016). Molecular cloning and characterization of two 1-deoxy-D-xylulose-5-phosphate synthase genes involved in tanshinone biosynthesis in *Salvia miltiorrhiza*. *Mol. Breed.* 36:124 10.1007/s11032-016-0550-3

