# Supplementary material for: Overexpression of LiDXS and LiDXR From Lily (Lilium ‘Siberia’) Enhances the Terpenoid Content in Tobacco Flowers
Source: Front Plant Sci. 2018 Jul 9;9:909. doi: 10.3389/fpls.2018.00909 (PMC6046550; doi:10.3389/fpls.2018.00909)
Supplement: Supplementary file 1 [file Presentation_1.pdf]

Supplementary Material

Overexpression of *LiDXS* and *LiDXR* from Lily (*Lilium* 'Siberia') Enhances the Terpenoid Content in Tobacco Flowers

Ming Sun*, Tengxun Zhang, Yanhong Guo, Xuejun Shi, Yongjuan Yang, Juntong Chen, Tangchun Zheng, Yu Han, Sagheer Ahmad

*** Correspondence:** Ming Sun: sunmingbjfu@163.com

1 Supplementary Figures and Tables

1.1 Supplementary Figures

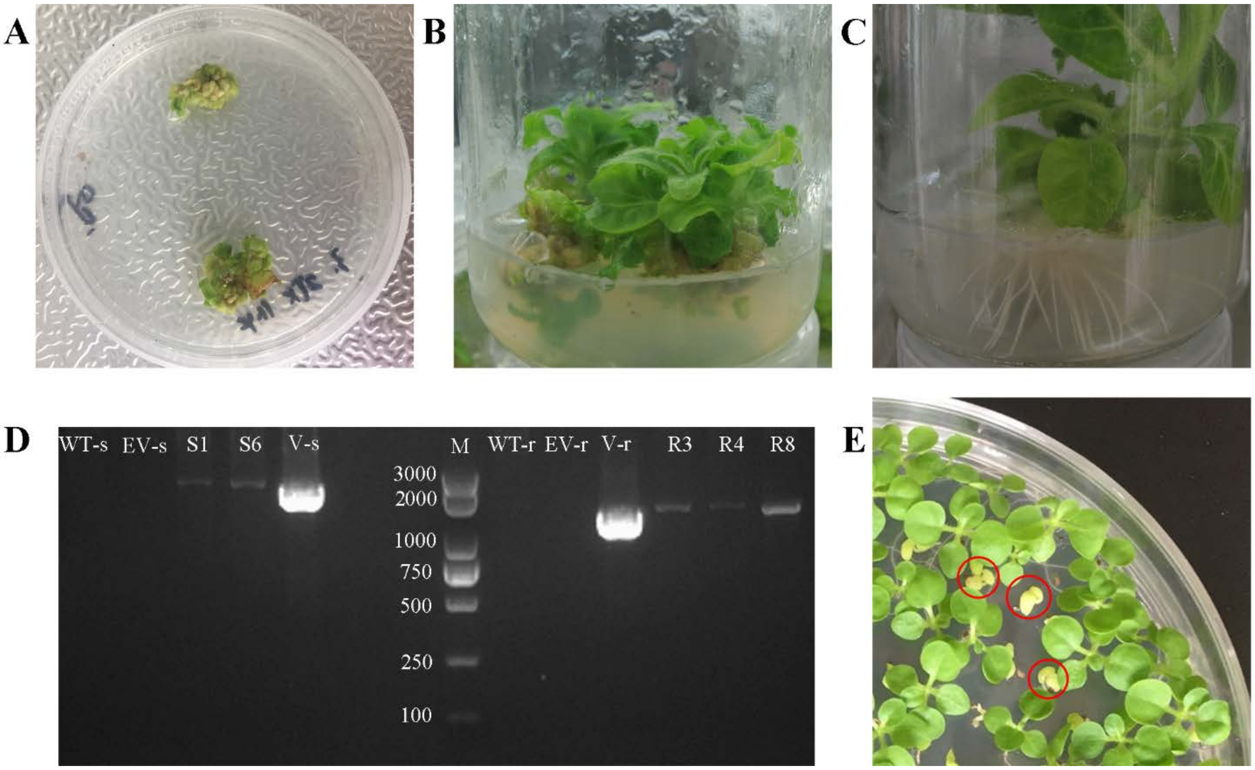

Supplementary Figure 1. The process of selection of transgenic plants. (A) Tobacco leaf differentiation. (B) Regeneration from callus. (C) Rooting culture. (D)WT: wild type, EV: transgenic lines with empty vector, M: DL3000 DNA Marker; V: expression vector, *pSuper1300::LiDXS* and *pSuper1300::LiDXR*. (E) Transgenic plants were screened on Murashige and Skoog's medium containing hygromycin. Sensitive plants are displayed in red cycles.

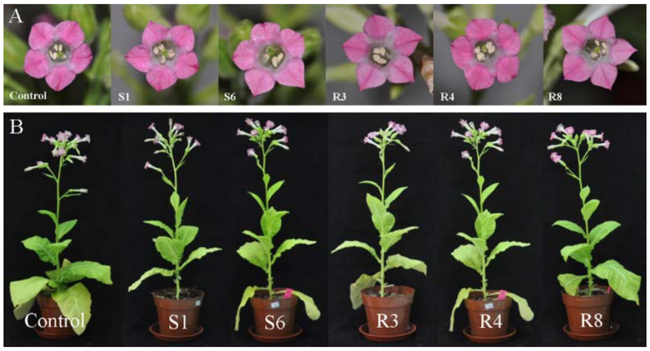

Supplementary Figure 2. The phenotypes of overexpressing transgenic plants. (A) The flower feature transgenic plants. (B) The phenotypes of whole plants.

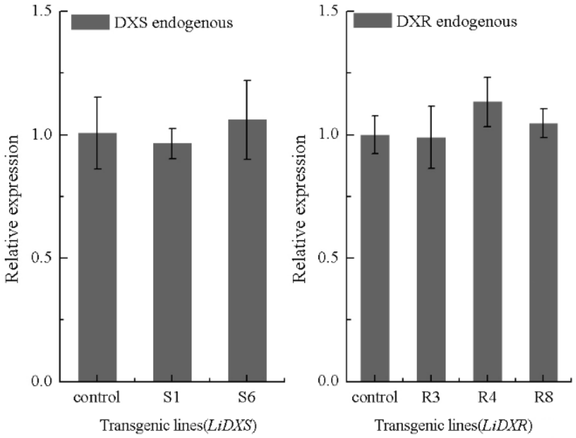

Supplementary Figure 3. Transcription profile of endogenous *DXS* and *DXR* genes in the flower of transgenic tobacco lines.

1.2 Supplementary Table

Supplementary Table 1. Primers sequences used in this study.

**Primers name**	**Sequence (5'→3')**	**Purpose**
DXS-F1	TTYGGCACSGGSCACAGYTC	intermediate fragment amplification
DXS-R1	GGRGCCATKACDAYCATGTT	
3' GSP	GCAAGATCAAGGATTTGCCAGCACCAGG	3' RACE
5' GSP	TCTTGCTGTCGACCTCCGCTTCTTTGA	5' RACE
DXS-F	ATGGGGGCCCTTCTCTTTCGTCAAG	full-length cDNA amplification
DXS-R	CTAACTCAAATGCATGGCCTCTTTC	
DXR-F	ATGGCAGCCCTGAAGCTTCCTCTAC	full-length cDNA amplification
DXR-R	TCATACAGGAACTGGACTCAAGCCA	
DXS-qF	AACAATCAGGTGTCTCTCCCGA	Real-time PCR
DXS-qR	GAACCGCCCTTCTCCTTTCTTATG	Real-time PCR
DXR-qF	ACCCAGATGCTGTCACGGTAGTTA	Real-time PCR
DXR-qR	GTGAGCAAGGGGAAGCACAAAG	Real-time PCR
NtEFα1- qF	ACACCCTGTACTACTCACTGAACG	Transgenic real-time PCR
NtEFα1- qR	GCAGTAGTGGTGAACGAGTAGCC	Transgenic real-time PCR
NtDXS- qF	CAACACAAACTTACTCAGGTCAAG	Transgenic real-time PCR
NtDXS- qR	TGCATAGTATTCTCCTCTTTCTGAC	Transgenic real-time PCR
NtDXR- qF	AGAACGAGACACACAGTACTTC	Transgenic real-time PCR
NtDXR- qR	AGTCCACAGGAGTAGGGAAA	Transgenic real-time PCR
DXS-pF	CCAAATCGACTCTAGAATGGGGGCCCTTCTCTTTCGT	Vector construction
DXS-pR	TACCGGATCCACTAGTCACTCAAATGCATGGCCTCTTTCGGC	Vector construction
DXR-pF	CCAAATCGACTCTAGAATGGCAGCCCTGAAGCTT	Vector construction
DXR-pR	TACCGGATCCACTAGTCTACAGGAACTGGACTCAAGCCA	Vector construction
